# Sexual dimorphism in the hypophysiotropic tyrosine hydroxylase-positive neurons in the preoptic area of the teleost, *Clarias batrachus*

**DOI:** 10.1186/s13293-015-0042-x

**Published:** 2015-11-09

**Authors:** Soham Saha, Saurabh Patil, Uday Singh, Omprakash Singh, Praful S. Singru

**Affiliations:** School of Biological Sciences, National Institute of Science Education and Research (NISER), Bhubaneswar, 751 005 Odisha India; Present address: Institut Pasteur, Affiliated to: Ecole des neurosciences Paris (ENP) Graduate program, 28, rue du docteur Roux, 75724 Paris, Cedex 15 France

**Keywords:** Dopamine, Nucleus preopticus periventricularis (NPP), Pituitary, Preoptic area, Sexual dimorphism, Reproduction, Teleosts

## Abstract

**Background:**

Dopamine (DA) neurons in the anteroventral periventricular nucleus (AVPV) in the preoptic area (POA) of mammals express estrogen receptors, regulate luteinizing hormone (LH) secretion, and show distinct sexual dimorphism. In teleosts, hypophysiotropic DA neurons of the nucleus preopticus periventricularis (NPP), located in the anteroventral POA, express estrogen receptors, innervate LH cells, and emerged as a neuroanatomical substrate for inhibiting LH cells. Interestingly, the NPP and AVPV seem to share several similarities. Whether DAergic neurons in the NPP show sexual dimorphism is, however, not known. Based on the proposed homology to AVPV and previous studies showing greater tyrosine hydroxylase (TH) mRNA and enzyme activity levels in the brain of female catfish, we hypothesize that females have greater number of DAergic neurons in the NPP and correspondingly more TH-immunoreactive fiber innervation of the pituitary.

**Methods:**

Adult, male and female *Clarias batrachus* collected during the prespawning phase of their reproductive cycle were used. Fish were anesthetized and perfused transcardially with phosphate-buffered saline (pH 7.4) and 4 % paraformaldehyde in phosphate buffer. Sections through the rostro-caudal extent of the POA and pituitary were processed for TH immunofluorescence. Using double immunofluorescence, the association between TH-immunoreactive fibers and LH cells in the pituitary was explored. Sections were analyzed using semiquantitative analysis.

**Results:**

NPP in POA of *C. batrachus* has two distinct subdivisions, viz, anterior (NPPa) and posterior (NPPp), and TH neurons were observed in both the subdivisions. Compared to that in the males, a significantly higher (*P* < 0.05) number of TH neurons was consistently observed in the NPPa of females. TH neurons in NPPp, however, showed no difference in the number or immunoreactivity. Since DA neurons in NPPa are hypophysiotropic, we compared TH-fiber innervation of the pituitary in both sexes. Compared to males, proximal pars distalis and LH cells in this region of the pituitary in females were densely innervated by TH fibers.

**Conclusions:**

Neurons of NPPa and their innervation to the pituitary seem to be a distinct sexually dimorphic DAergic system in *C. batrachus*. The DAergic system may serve as a component of the neural mechanisms controlling the sexually dimorphic LH surge in teleosts. Given the similarities shared by NPPa and AVPV, homology between these two nuclei is suggested.

## Background

Similar to mammals, the preoptic area (POA) also plays a key role in the neuroendocrine regulation of reproduction in teleosts [1, 2]. The neurons of the nucleus preopticus periventricularis (NPP) residing in the anteroventral POA has emerged as an important component of the neuroendocrine mechanisms regulating luteinizing hormone (LH) cells and reproduction in teleosts. Using neuronal tracing, neurons in the NPP were shown to innervate the pituitary (*Salmo salar*: [3], *Carassius auratus*: [4], *Clarias batrachus*: [5, 6]). Two subdivisions of the NPP, viz, anterior (NPPa) and posterior (NPPp), have been identified in teleosts [7–9]. While both subdivisions contain tyrosine hydroxylase [TH, a marker for dopamine (DA)] neurons, TH neurons in the NPPa are hypophysiotropic [7, 8]. The inhibitory DA neurons in POA seem directly responsive to estradiol and may serve as principal mediators of the negative feedback [10]. This is supported by the presence of gonadal steroid receptors in TH neurons of the anteroventral POA of the rainbow trout, *Oncorhynchus mykiss* [11, 12], and changes in TH messenger RNA (mRNA) expression in these neurons of the European eel, *Anguilla anguilla*, following castration and testosterone treatment [13]. DA neurons in the NPPa seem important in the regulation of LH cells and reproduction since lesion of the rostral POA containing NPP resulted in a loss of inhibitory control on LH secretion in the goldfish, *C. auratus* [14, 15]. A recent study on midshipman fish, *Porichthys notatus*, supports the role of DA neurons in NPPa as inhibitory regulators of reproduction [16]. In this fish, greater number of TH neurons was observed in the anterior parvocellular preoptic nucleus of non-reproductive females compared to those of the reproductive females [16]. The other sub-population of TH neurons in the NPPp of *C. batrachus* and *Cirrhinus cirrhosus* seems non-hypophysiotropic [7, 8].

The mechanisms regulating LH surge in teleosts have been suggested to be sexually dimorphic [17, 18], but the relevance of the hypophysiotropic DAergic system in these pathways has remained elusive. While Kawabata et al. [19] observed sexual dimorphic TH expression in the optic tectum of medaka, *Oryzias latipes*, the DA levels and DA transporter mRNA expression in the brain of zebrafish, *Danio rerio*, did not show any sexual dimorphism [20]. Although sex differences in the TH transcript and TH enzyme activity have been demonstrated in the brain of two species of catfish, *C. batrachus* [21] and *Heteropneustes fossilis* [22], sexually dimorphic DAergic neurons in the POA and their targets in the pituitary is unexplored. DA neurons in the anteroventral periventricular nucleus (AVPV) of mammals express estrogen receptors [23], show distinct sexual dimorphism [24], and play an important role in transducing the hormonal signals to LH cells and ovulation [25, 26]. While TH neurons were observed in two distinct subdivisions of NPP in *C. batrachus* [7], those in NPPa seem to share several similarities with AVPV (Table 1). We hypothesize that the DA neurons in the NPPa are homologous to DA neurons in the AVPV and that females have a greater number of DA neurons in the NPPa and more TH-immunoreactive (TH-ir) fibers innervating the proximal pars distalis (PPD) of the pituitary. Male and female *C. batrachus* collected during prespawning phase of the reproductive cycle were employed to measure differences in number of DA neurons and their innervation to the pituitary between sexes. The information about neuroanatomical organization of the forebrain including POA, distribution of neuropeptides like GnRH and neuropeptide Y, their role in regulation of LH cells [27–29], and the organization of hypophysiotropic and non-hypophysiotropic DA neurons in POA is available in this fish [7]. It seems appropriate to undertake this study in the prespawning phase since the TH enzymatic activity in the telencephalon and hypothalamus of *H. fossilis* attains peak during this phase [22]. Using immunofluorescence, we compared the number of TH**-**ir neurons in the NPP of male and female *C. batrachus*. The LH cells reside in the PPD of the pituitary [27], and these cells are regulated by the DAergic neurons originating from the anteroventral preoptic region [15, 30]. While lesion of the anteroventral POA resulted in the disappearance of DA fibers in the pars distalis in *C. auratus* [15], retrograde neuronal tracing suggests that the DA neurons in this region of *C. batrachus* are hypophysiotropic [7]. We therefore determined if TH-ir fibers in the PPD of *C. batrachus* show sexual dimorphism.

**Table 1:** Similarities between NPPa of teleosts and mammalian AVPV

	NPPa^a^	AVPV
Neuroanatomical location	Anteroventral preoptic area, around the preoptic recess [2, 7, 31, 38].	Ventral position in the periventricular zone of the preoptic nucleus [68].
Dopamine (tyrosine hydroxylase) neurons	Contain several DA neurons [7, 8, 12, 13, 39, 47] and serve as an important DAergic neuronal group involved in the regulation of gonadotropin secretion and reproduction [2, 15, 37, 39].	Contain several neurons and serve as a nodal component of neural circuitry controlling gonadotropin secretion [68].
Estrogen receptor (ER) expression	Presence of ERα as well as ERβ expression was observed [11, 12, 69, 70].	Contains both ERα and ERβ [23, 74, 75].
TH neurons expressing ER	Several TH neurons co-express ER [11, 12].	Harbors TH neurons expressing ERα [75] and ERβ [23].
Sexually dimorphic TH neuronal number	Higher in females compared to that in males (present study).	Higher in females compared to that in males [23, 24, 76].
Kisspeptin-containing system	*kiss 2* neurons were seen in the POA [71] at a similar location to the NPOav, and these neurons seem to co-express ER [72]. POA *kiss* neurons are regulated by gonadal steroids [82; 83].	Harbors *kiss 1* neurons [78]; these neurons co-express ER and are regulated by gonadal steroids [79].
Cocaine- and amphetamine-regulated transcript (CART) neurons and fibers	Few CART cells but several fibers [73].	Few CART cells and dense CART innervation [77].
NPY neurons and fibers	Few NPY cells and several fibers [8].	NPY cells and fibers were seen in and around AVPV [80].

^a^NPPa of *C. batrachus* corresponds to the anterior parvocellular preoptic nucleus (PPa) [46, 47], nucleus preopticus pars anteroventralis (NPOav) [12, 13, 38], anteroventral part of the parvocellular preoptic nucleus (NPOav), nucleus preopticus periventricularis [81], preoptico-hypophyseal dopaminergic (POHDA) neurons [51], and part of the nucleus preopticus parvocellularis anterioris [9] in the anteroventral POA of other teleosts

## Methods

### Animals and tissue processing

Adult, males [body weight (BW) 102 ± 8.2 g; standard length (SL) 23.3 ± 1.5 cm; gonadosomatic index (GSI) 2.4 ± 0.24] and females [BW 112 ± 15.5 g; SL 25.4 ± 2.0 cm; GSI 8.3 ± 0.30] *C. batrachus* were collected from the local ponds around Bhubaneswar, India, during May and June, which corresponds to the prespawning phase of their reproductive cycle [27]. Fish were maintained under the normal photoperiod and temperature for 48 h and fed with small pieces of goat liver. All the experimental procedures were approved by the Institutional Animal Ethical Committee (IAEC) at NISER, Bhubaneswar, under the Committee for the Purpose of Control and Supervision of Experiments for Animals (CPCSEA), New Delhi, India.

Male and female *C. batrachus* (*n* = 6, per group) were anesthetized using 2-phenoxyethanol (Sigma, dilution 1:2000). The fish were transcardially perfused with 100 ml phosphate-buffered saline (PBS; pH 7.4) followed by 100 ml of 4 % paraformaldehyde in 0.1 M phosphate buffer (PB). Brains along with the pituitary gland were dissected out and post-fixed in the same fixative overnight at 4 °C. The brain and pituitary were cryoprotected by immersing in 25 % sucrose solution in PBS overnight at 4 °C, embedded in tissue mounting media, and cut serially in a transverse plane (20-μm thickness) on a cryostat (CM3050S, Leica, Germany). Sections were collected in PBS for immunofluorescence labelling as described below.

### Immunofluorescence

Sections through the rostro-caudal extent of the POA and hypothalamus of the male and female *C. batrachus* were processed for TH immunofluorescence as described previously [7]. Sections were rinsed in PBS and immersed in 0.5 % Triton X-100 in PBS for 20 min to improve the antibody penetration. Sections were incubated in blocking solution (5 % normal horse serum and 0.1 % Triton X-100 in PBS) for 30 min followed by rabbit polyclonal TH antiserum (Millipore, Cat. # AB152; dilution 1:1000) for 12 h at 4 °C. Following rinsing in PBS, sections were incubated in Cy3-conjugated goat anti-rabbit IgG (Jackson Immunoresearch; dilution 1:500) for 4 h at room temperature. Sections were rinsed in PBS and in Tris buffer (pH 7.6), mounted with Vectashield mounting medium containing 4',6-diamidino-2-phenylindole (DAPI; Vector), and observed under the AxioImager M2 fluorescence microscope (Carl Zeiss, Germany). To determine whether (i) TH fibers in PPD and (ii) TH innervation of LH cells of *C. batrachus* show sexual dimorphism, transverse sections of the pituitary of male and female *C. batrachus* were processed for TH immunofluorescence as described above or TH/LH double immunofluorescence as given below.

Sections were prepared for immunofluorescence and incubated overnight at 4 °C in a mixture of rabbit polyclonal human LH-β antiserum (National Hormone and Pituitary Program, NIH, USA; dilution 1:500) and sheep polyclonal TH antiserum (Millipore, Cat. # AB1542; dilution 1:500) in antibody diluent. Sections were rinsed in PBS and incubated in a mixture of Cy3-conjugated anti-rabbit IgG (1:500) and Alexa Fluor 488-conjugated anti-sheep IgG (Invitrogen, 1:500) for 4 h at room temperature. The sections were rinsed in PBS, mounted onto glass slides with Vectashield, and observed under an AxioImager M2 fluorescence microscope.

All the experimental conditions including buffers, fixative, thickness of the sections, time and temperature of the incubation, and dilution of the antibodies were kept constant for processing of the brains and pituitary. Sections of the POA containing NPP from all the animals were processed together to minimize the error. Similarly, pituitary gland sections from all animals were processed together. Specificity of the TH antiserum in POA and human LH-β antiserum in the PPD of *C. batrachus* has already been demonstrated [7]. Sections were observed under a fluorescence microscope, and images were captured. All the images were equally adjusted for the size and brightness using Adobe Photoshop CS4 software (Adobe Systems Inc., CA), and panels were prepared. Schematic drawings of the POA were adopted from Singh et al. [7] and Rama Krishna and Subhedar [31] to diagrammatically represent the TH neurons in POA of *C. batrachus*. Different subdivisions of the pituitary of *C. batrachus* were identified as described previously [27].

### Semiquantitative analysis

Transverse sections through the POA around the preoptic recess (POR) of each animal were analyzed under the AxioImager M2 fluorescence microscope. TH-immunoreactive (TH-ir) neurons in the POA of *C. batrachus* extend rostro-caudally. While TH-ir neurons in the NPPa spread anteroventrally in the POA, those in the NPPp are located close to the paraventricular subdivision of the nucleus preopticus (NPOpv) and extend dorsally [7]. Neurons of NPOpv are located such that they are flanked anteriorly by the TH-ir neurons of NPPa and posteriorly by NPPp [7]. Sections at the level of the anterior commissure and POR until the appearance of NPOpv encompass NPPa whereas, in caudal sections, the TH-ir neurons residing ventral to NPOpv are demarcated as NPPp. For counting the TH-ir cells in NPP, ten sections through the rostro-caudal extent of POA containing NPPa and NPPp were analyzed. Neurons with distinct TH immunofluorescence and nucleus, as visualized with DAPI, were counted for each male and female fish. The cell numbers were corrected using Abercrombie’s method [32] by taking into account the thickness of the section, actual profile count, and mean nuclear diameter. The data from the male and female fish was pooled separately, and the mean ± SEM was calculated.

To determine if there is a sexually dimorphic pattern of TH-ir fiber innervation in the PPD, four fluorescently labelled alternate pituitary sections from each animal were analyzed using image analysis as described earlier [7, 8]. The sections were sampled serially to cover the rostro-caudal extent of PPD in each animal. Intensity of TH-ir and percent fluorescent area of TH-ir in PPD were measured. Briefly, the image analysis system consisted of a fluorescence microscope (Carl Zeiss, Germany) and a CCD camera. During analysis, the objective and exposure as well as adjustments in the image acquisition software were kept at constant settings. The slides were coded, and an observer blind to the experiment analyzed the slides. For measuring the TH-ir intensity, images were captured and analyzed using Zen2011 software (Carl Zeiss). The pixel intensities were taken from the PPD of each animal, and the background intensity of the non-immunoreactive area in the PPD was subtracted from the intensity of immunoreactivity. The data from all male and female fish was separately pooled and represented as mean ± SEM. The difference in mean intensity of TH-ir in the PPD of males and females was statistically analyzed.

Percent fluorescent area of TH-ir fibers in PPD was measured as described earlier [33]. Keeping the settings of the microscope constant, images were captured. Sections were imaged and analyzed using Image J software. After thresholding, the area occupied by TH-ir fibers in PPD was measured in all the fish. The data from each fish was separately pooled and averaged, and the mean ± SEM was determined. The difference in percent fluorescent area of TH-ir fibers in the PPD of males and females was statistically analyzed. To determine whether the association between TH-ir fibers and LH cells in the PPD show sexual dimorphism, four double-labelled sections through the rostro-caudal extent of the PPD from each male and female *C. batrachus* were analyzed under the fluorescence microscope. The method has already been described [7]. By switching the filter sets (red for Cy3 and green for Alexa Fluor 488), the number of LH cells contacted or not contacted by the TH-ir fibers was counted for each animal and percentage was determined. The data was pooled, and the mean ± SEM was calculated.

### Statistical analysis

The data analysis was performed using Prism (GraphPad Software, Inc., CA) and R statistical software. All the tests performed were two-tailed. Shapiro-Wilk test was used to assess normality of the data. Due to non-normal distribution, the data was analyzed using non-parametric Mann-Whitney test. To determine whether or not the number of TH-ir neurons in the NPP and their fiber innervation to the PPD have any correlation with body size, we used Pearson’s correlation between BW/SL and either TH-ir neuron number in the NPP or TH-ir fiber data in the PPD. For the statistical analysis, *P* < 0.05 was considered as statistically significant.

## Results

**Fig. 1:**
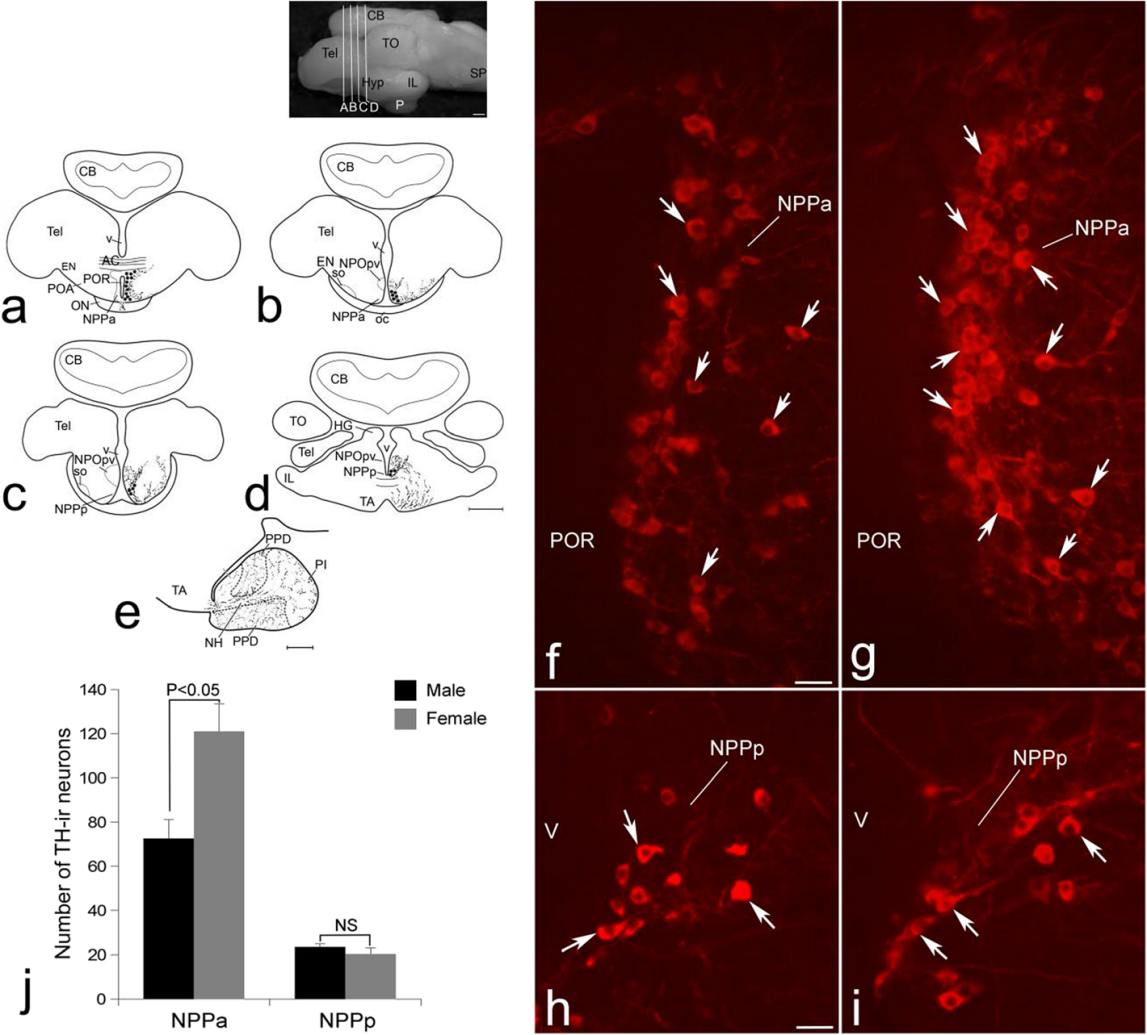
Schematic of the transverse sections through the forebrain (**a**–**d**) and sagittal section of the pituitary gland (**e**) of *Clarias batrachus* showing the organization of tyrosine hydroxylase (TH) immunoreactive (ir) cell bodies (*filled circles*) and fibers (*dashed lines and dots*). TH cell bodies are seen in the anterior (NPPa) and posterior (NPPp) subdivisions of the nucleus preopticus periventricularis (NPP). The photomicrograph of the brain of *C. batrachus* at the top shows the levels of transverse sections used for depicting the TH immunoreactivity. In the pituitary gland, a dense innervation of TH-ir fibers is seen in the proximal pars distalis (PPD). Immunofluorescence photomicrographs through the POA of male (**f**, **h**) and female (**g**, **i**) *C. batrachus* showing TH-ir neurons (*arrows*) in the NPPa (**f**, **g**) and NPPp (**h**, **i**). **j** Semiquantitative image analysis of the number (mean ± SEM) of TH neurons in the NPPa and NPPp in both the sexes. Note the presence of more number of TH-ir neurons in the NPPa of female fish. *P* < 0.05. *NS* non-significant. Scale bar = 1 mm in (**a**–**d**) and the photomicrograph of the brain at top , 500 μm in (**e**), and 25 μm in (**f**–**i**)

Organization of TH-ir neurons in the POA of *C. batrachus* has already been described [7]. Briefly, in POA, the TH-ir neurons were located in the nucleus preopticus periventricularis (NPP) on either side of the POR (Fig. 1). NPP consists of two subdivisions, viz, NPPa (Fig. 1a, b) and NPPp (Fig. 1c, d). While TH-ir neurons of NPPa are distributed anteroventrally around the POR (Fig. 1a, b), those in NPPp were located ventral to the NPOpv (Fig. 1c, d). TH-ir fibers were observed around the POR, innervating the nucleus preopticus (NPO) and tuberal region (Fig. 1a–d). The pituitary gland of *C. batrachus* showed three distinct subdivisions, viz, proximal pars distalis (PPD), rostral pars distalis (RPD), and pars intermedia (PI) (Fig. 1e). TH fibers were seen in all three regions of the pituitary gland (Fig. 1e).

TH-ir neurons and fibers were observed in both subdivisions of the NPP in the male as well as female *C. batrachus* (Fig. 1f–i). A distinct difference in the number of TH-ir neurons was seen in the NPPa of males and females (Fig. 1f, g). Significantly higher (*P* < 0.05) number of TH-ir neurons was observed in the NPPa of females compared to that in males (Fig. 1f, g, j). No significant difference in the number of TH-ir neurons was observed in the NPPp between males and females (Fig. 1h–j).

**Fig. 2:**
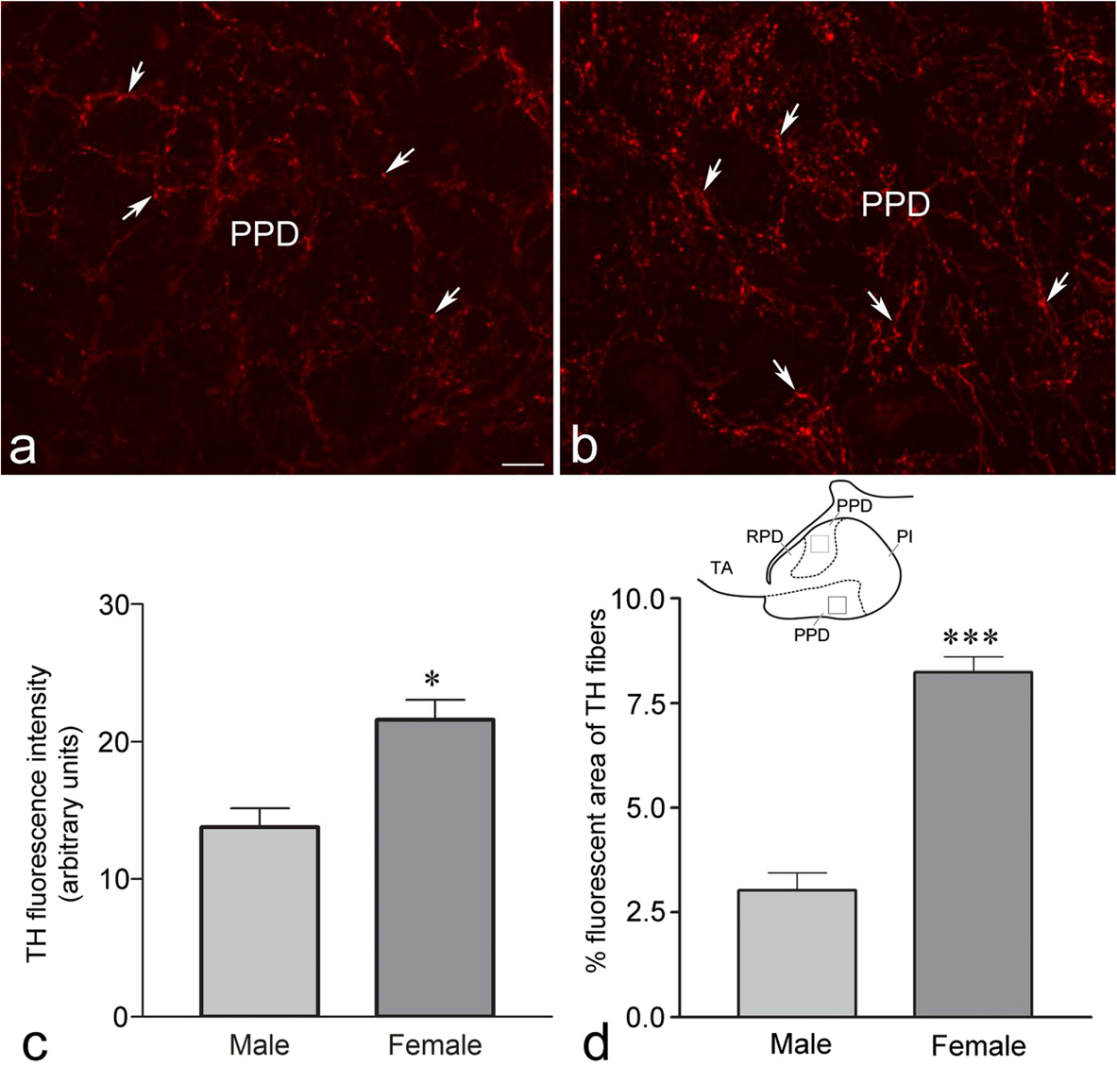
Immunofluorescence photomicrographs of sections through the proximal pars distalis (PPD) of the pituitary of the male (**a**) and female (**b**) *Clarias batrachus* showing tyrosine hydroxylase (TH) immunoreactive (ir) fibers (*arrows*). Semiquantitative image analysis of the (**c**) intensity of TH-ir and (**d**) percent fluorescent area occupied by TH-ir fibers in the PPD of the male and female. *Open squares* in the inset indicate the areas from where the measurements were collated. Note the significantly more TH-ir fibers in the PPD of female fish. *P < 0.05 and ***P < 0.0001 compared to males. Scale bar = 25 μm

**Fig. 3:**
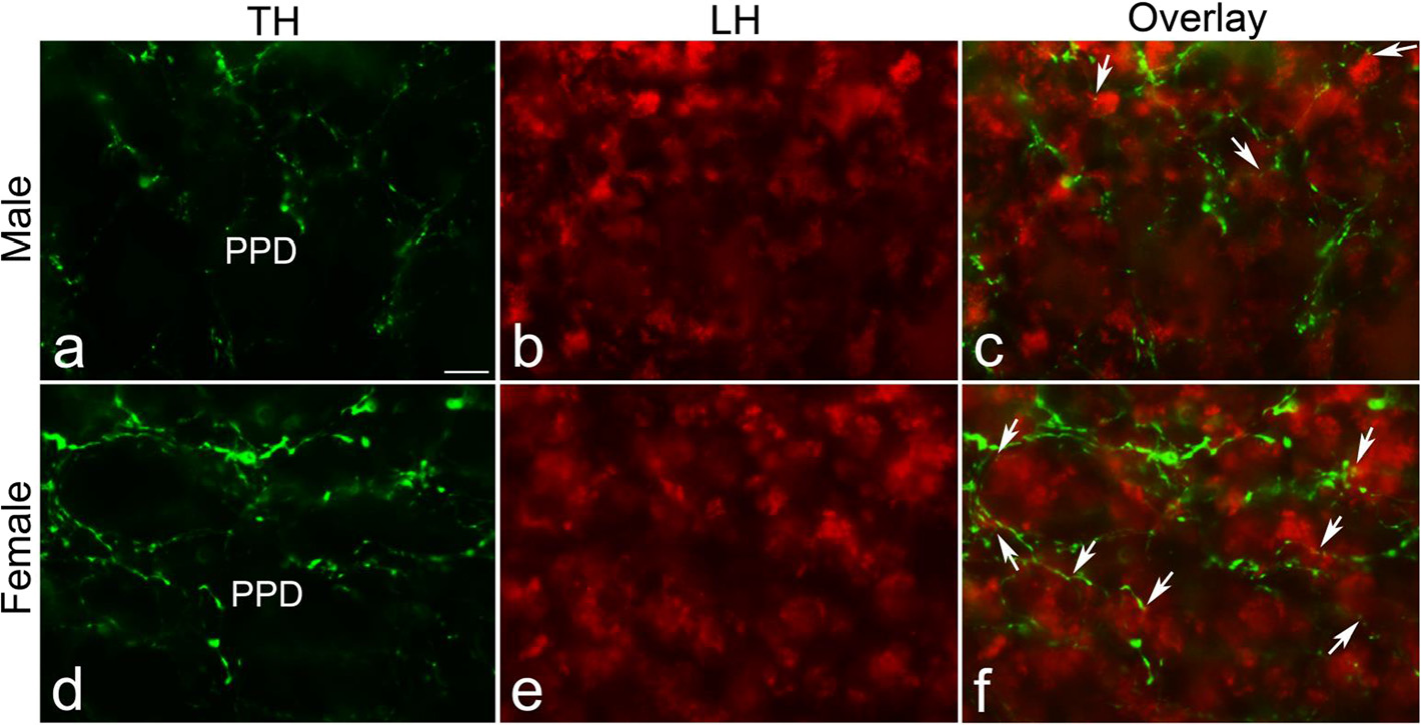
Double immunofluorescence photomicrographs of transverse sections through the proximal pars distalis (PPD) of the pituitary of the male (**a**–**c**) and female (**d**–**f**) *Clarias batrachus* showing the association between tyrosine hydroxylase immunoreactive (TH-ir) fibers (*green*) and LH cells (*red*). LH cells contacted by TH-ir fibers are shown with *arrows*. Greater number of LH cells is contacted by TH-ir fibers in the PPD of females. Scale bar = 25 μm

DA regulates LH cells, and these cells reside in the PPD of the pituitary [7, 27]. Since females showed a greater number of TH-ir neurons in NPPa, we investigated the TH-ir fiber innervation in the PPD of the pituitary, which is known to be one of the targets of NPPa DA neurons. Compared to the TH-ir fibers in PPD of males (Fig. 2a, c, d), the PPD of females showed a significantly greater intensity of TH immunofluorescence (Fig. 2b, c; *P* < 0.05) and percent fluorescent area of TH-ir fibers (Fig. 2b, d; *P* < 0.0001). The number of TH-ir neurons in the NPP and TH-ir fibers in the PPD showed no significant (*P* > 0.05) correlation with body size. While LH cells in the PPD were contacted by TH-ir fibers in both sexes, a higher percentage of LH cells contacted by TH-ir fibers was observed in the PPD of females than those in males (males: 38.13 ± 3.81 % and females: 74.6 ± 3.06 %) (Fig. 3).

## Discussion

Unlike in tetrapods, the median eminence is absent in teleosts and the hypophysiotropic neurons directly innervate and regulate pituitary cells [34]. Two potent neuromodulators, GnRH and DA, orchestrate the central regulation of LH cells in teleosts. The LH cells are innervated by DA as well as GnRH axons [30] and act via their distinct receptors [35, 36]. While DA exerts a potent inhibitory tone on LH cells and reproduction in teleosts, GnRH is stimulatory [10, 37, 38]. DA neurons residing in the anteroventral POA inhibit LH cells and reproduction in teleosts [15]. DA neurons in the NPPa, residing in the anteroventral POA, project to the pituitary [7, 39], whereas those in the NPPp are non-hypophysiotropic [7]. In the present study, we noticed a distinct sexual dimorphism in the number of TH neurons in the NPP. While higher number of TH-ir neurons was observed in NPPa of female *C. batrachus*, those in NPPp were comparable in both sexes. Although several studies have reported the sexual dimorphic expression of neurotransmitters/neuromodulators in the POA of teleosts [40–44], relevant information about DA is inconclusive. Except for the optic tectum, no difference in TH mRNA expression was observed in the brains of male and female medaka, *O. latipes* [19]. Studies hint at sexual dimorphism in POA dopaminerergic neurons (Adrio and Kah, in Le Page et al. [45]). While qRT-PCR analysis of the brain tissues of *C. batrachus* showed twofold more abundance of the *Th* transcript in the female brain compared to the male brain [21], measuring TH mRNA in the entire brain could not resolve the region-specific expression and the neuronal group exhibiting specific change. In the present study, we have identified a distinct sexually dimorphic subpopulation of TH-ir neurons in POA, their hypophysiotropic projections, and association with LH cells in a teleost *C. batrachus*. The presence of more number of TH-ir neurons in NPPa of the female *C. batrachus* is in agreement with the sexually dimorphic DAergic neuronal population in the mammalian AVPV [23, 24].

While several studies explored the organization of TH-ir- or mRNA-expressing neurons in the POA of teleosts, different nomenclature was used to describe these neurons in different teleosts. Mainly, three terminologies, viz, (i) anterior (PPa) and posterior (PPp) parvocellular preoptic nuclei (*Apteronotus leptorhynchus*: [46], *D. rerio*: [47]), (ii) nucleus preopticus pars anteroventralis (NPOav) (*O. mykiss*: [11, 12, 48], *A. anguilla*: [13]), and (iii) NPPa and NPPp (*Tinca tinca*: [49], *C. batrachus*: [7], *C. cirrhosus*: [8]), seem widely used to describe the POA DAergic neurons in teleosts. In *Solea senegalensis*, the preoptic TH-ir neurons were observed in the anteroventral part of the parvocellular preoptic nucleus (NPOav) and posterior periventricular nucleus (NPPv) [50]. Recently, the terminology “preoptico-hypophyseal dopamine (POHDA)” neurons was used to describe the TH-ir neurons projecting to the pituitary gland of zebrafish [51].

DA neurons in the POA of teleosts express gonadal steroid receptors and may serve as principal mediators of negative feedback [10]. While the TH-ir neurons in NPOav of *O. mykiss* express estrogen receptors (ER) [11], using *in situ* hybridization histochemistry, Weltzien et al. [13] observed that the TH mRNA expression in NPOav neurons of *A. anguilla* was modulated by treatment with testosterone. Given the hypophysiotropic nature of DA neurons and presence of ER, a direct DAergic inhibition of the LH cells by gonadal steroids is suggested. Information about the sexually dimorphic expression/levels of DA in the brain of teleosts is available, but the reports are contradictory. In *O. latipes*, the expression of *th1* and *th2* mRNA in various brain regions was examined using real-time PCR as well as in situ hybridization [19]. While no sexual dimorphism was seen in the *th1* and *th2* expression using *in situ* hybridization, real-time PCR analysis showed a slightly higher expression of *th2* in the optic tectum of females [19]. In *D. rerio*, brain DA levels measured using HPLC and DA transporter mRNA expression analyzed with quantitative real-time PCR did not show any sexual dimorphism [20]. In the catfish, *H. fossilis*, higher TH enzyme activity was observed in the telencephalon, hypothalamus, and medulla oblongata of females [22]. Although two non-allelic *Th* genes are reported in teleosts, a single transcript of *Th* was observed in the brain of *C. batrachus* [21]. The full-length cDNA of *Th* was cloned, and its expression in the brain of male and female *C. batrachus* was studied [21]*.* Compared to males, the female brain contains a high copy number of TH mRNA [21]. While these studies analyzed difference in TH levels in the entire brain, the present study has identified a distinct sexually dimorphic population of TH-ir neurons in the POA of *C. batrachus*.

*C. batrachus* is a seasonal breeder, and the present study was conducted during the prespawning phase of its reproductive cycle. The circulating levels of estradiol in *C. batrachus* [52] and TH levels in the telencephalon/hypothalamus of another coexisting catfish, *H. fossilis* [22], remain higher in females during the spawning phase than other phases of their reproductive cycle. The greater number of TH-ir neurons in NPPa, more intensity and percent area occupied by TH-ir fibers in PPD, and more LH cells contacted by TH-ir fibers in PPD might be due to the higher estradiol levels during the prespawning phase of the reproductive cycle of this fish. However, in another teleost, *P. notatus*, greater number of TH-ir neurons was observed in the POA of non-reproductive compared to the reproductive females [16]. We speculate that the sexually dimorphic pattern of TH-ir in the POA and pituitary of *C. batrachus* might be restricted to a specific season, and the fish collected during other phases of the reproductive cycle may show a lack of sex difference. It is, however, important to note that the sexual dimorphism in TH enzymatic activity in the telencephalon/hypothalamus of *H. fossilis* was observed throughout the reproductive cycle with higher TH levels in the female brain compared to the male brain [22]. The TH-ir neurons in NPPa of *H. fossilis* may also exhibit sexual dimorphism as observed in *C. batrachus*.

Our results are comparable to the sexually dimorphic pattern of TH mRNA expression and TH enzymatic activity in the brain of *C. batrachus* and *H. fossilis*, respectively, as well as with the information available in mammals. Compared to males, the AVPV of female rats contained three to four times more TH-stained perikarya [24]. While the functional significance of the presence of higher TH-ir neurons in NPPa and dense TH-ir fiber innervation of the pituitary in females is not known, we suggest that the regulation of LH cells in the pituitary of both sexes might be differentially regulated. The mechanisms regulating preovulatory LH surge in the goldfish seem sexually dimorphic, and different pathways regulating LH secretion in males and females have been suggested [17]. The number of TH-ir neurons in NPPp in the POA of male as well female *C. batrachus*, however, was comparable. TH-ir neurons in NPPp are non-hypophysiotropic in nature [7], and whether these neurons express gonadal steroid receptors is unknown.

In the rat brain, the catecholaminergic cell populations have been categorized as A1–A17 and are located from the medulla oblongata to the hypothalamus [53–55]. DA neurons of the A11–A15 groups are located in the diencephalon [54, 56]. While the A12 group resides in the arcuate nucleus, A15 DAergic neurons are located in the AVPV [57]. Although the DA neurons in the PPa of teleosts and the mammalian A12 group differ in their projection patterns [58], both seem to negatively regulate LH cells [54]. A similarity between these DAergic cell groups is therefore suggested [54]. Linard et al. [11] compared the preoptic ER/TH neurons in *O. mykiss* with the mammalian DAergic neuronal groups and suggested that the ER-expressing TH neurons of NPOav is a characteristic of teleost fish. Recently, a comparison between DAergic cell groups in the zebrafish and rat brain suggests a similarity between DA neurons in the PPa of zebrafish with the A14 and A15 DAergic groups [59]. In view of the location of the NPP neurons in the anterior preoptic area around the ventricle, expression of ER, and distinct sexual dimorphism similar to AVPV, we suggest that the NPPa DAergic neurons may be considered similar to the A15 DAergic group. This is in agreement with earlier studies [59–61]. Although determining one-to-one homology between DAergic groups among vertebrates seems difficult [54], similarities shared between DA neurons in AVPV and NPPa may suggest that these two nuclei are homologous.

## Conclusions

Similar to AVPV, the presence of a well-characterized DAergic neuronal population in the POA of teleosts showing distinct sexual dimorphism has remained unexplored [45]. The present study demonstrates that the TH-ir neurons in NPPa and their fibers supplying the PPD exhibit a distinct sexual dimorphism with higher number in females compared to those in males. The sexually dimorphic organization of the DAergic neurons controlling LH cells and their hypophysiotropic projections might be a crucial component of the neural mechanism controlling differential LH surge. In addition, the greater number of DA cells in the NPPa of females may be attributed to the inherent anatomical and physiological features of the ovary. The GSI of females in the prespawning and spawning phases is greater than that in the males, and there is relatively more pressure created by the growing mass of the ovaries compared to the testes on the gonad wall. Increasing GSI from the prespawning through spawning phases [52] and build up of intra-ovarian pressure following maturation of oocytes are believed to activate stretch pathways in the ovarian wall, which may impinge on neurons of NPO in the POA of *C. batrachus* [62, 63]. While neurons of NPO in teleosts synthesize isotocin (a homolog of mammalian oxytocin), administration of the fish neurophysial hormones/synthetic oxytocin initiates spawning reflex in the killifish, *Fundulus heteroclitus* [64]. Additionally, isotocin fibers of NPO innervate the pituitary of *C. auratus* [65], contact LH cells of *C. batrachus* [7] and *Dicentrarchus labrax* [66], and regulate LH cells [7]. Isotocin has a potent stimulatory effect on LH secretion since administration of the peptide resulted in a significant increase in the serum LH levels in goldfish [67]. We speculate that due to increase in intra-ovarian pressure, isotocin neurons of NPO may get activated and in turn stimulate LH cells. The female spawns during July–August which coincides with the arrival of monsoon rains. Having more DAergic neurons in NPPa of females may provide additional inhibitory influence on LH cells and might be necessary to overcome the intra-ovarian pressure-induced activation of NPO neurons and premature LH release and initiation of the spawning reflex.

The similarities shared between NPPa in teleosts and AVPV in mammals, as given in Table 1, may help us to draw homology between these two nuclei. Both these nuclei occupy comparable neuroanatomical positions in POA and contain several similar neuromodulator-containing cell bodies. TH-ir neurons in AVPV [23, 24] and those in the NPOav of rainbow trout [10, 11] co-express ER. TH-ir neurons in the AVPV show sexual dimorphism. The AVPV of female rats contain more TH-ir neurons than those in the male [23, 24] and is comparable to the TH-ir neurons seen in the NPPa of *C. batrachus*. Taking into consideration the similarities shared between NPPa and AVPV, we propose that the nucleus might be homologous with mammalian AVPV.

## Abbreviations

AC: anterior commissure
CB: cerebellum
EN: entopeduncular nucleus
HG: habenular ganglion
Hyp: hypothalamus
IL: inferior lobe of hypothalamus
NH: neurohypophysis
NPO: nucleus preopticus
NPOpv: NPO, paraventricular subdivision
NPOso: NPO, supraoptic subdivision
NPPa: nucleus preopticus periventricularis anterior subdivision
NPPp: nucleus preopticus periventricularis posterior subdivision
oc: optic chiasm
ON: optic nerve
P: pituitary
PI: pars intermedia
POA: preoptic area
POR: preoptic recess
PPD: proximal pars distalis
RPD: rostral pars distalis
SP: spinal cord
TA: tuberal area
Tel: telencephalon
TO: optic tectum
V: ventricle
